# BAP1-Related ceRNA (NEAT1/miR-10a-5p/SERPINE1) Promotes Proliferation and Migration of Kidney Cancer Cells

**DOI:** 10.3389/fonc.2022.852515

**Published:** 2022-03-29

**Authors:** Rui-ji Liu, Zhi-Peng Xu, Shu-Ying Li, Jun-Jie Yu, Ning-han Feng, Bin Xu, Ming Chen

**Affiliations:** ^1^ Department of Urology, Affiliated Zhongda Hospital of Southeast University, Nanjing, China; ^2^ Surgical Research Center, Institute of Urology, Southeast University Medical School, Nanjing, China; ^3^ Sichuan Cancer Hospital & Institute, Sichuan Cancer Center, Cancer Hospital affiliate to School of Medicine, UESTC, Chengdu, China; ^4^ Department of Urology, Wuxi No.2 People’s Hospital of Nanjing Medical University, Wuxi, China; ^5^ Nanjing Lishui District People’s Hospital, Zhongda Hospital Lishui Branch, Southeast University, Nanjing, China

**Keywords:** ceRNA, DNA methylation, immune microenvironment, clear-cell renal cell carcinoma, prognosis

## Abstract

**Background:**

*BAP1* is an important tumor suppressor involved in various biological processes and is commonly lost or inactivated in clear-cell renal cell carcinoma (ccRCC). However, the role of the BAP1-deficient tumor competing endogenous RNA (ceRNA) network involved in ccRCC remains unclear. Thus, this study aims to investigate the prognostic BAP1-related ceRNA in ccRCC.

**Methods:**

Raw data was obtained from the TCGA and the differentially expressed genes were screened to establish a BAP1-related ceRNA network. Subsequently, the role of the ceRNA axis was validated using phenotypic experiments. Dual-luciferase reporter assays and fluorescence *in situ* hybridization (FISH) assays were used to confirm the ceRNA network.

**Results:**

Nuclear enriched abundant transcript 1 (NEAT1) expression was significantly increased in kidney cancer cell lines. NEAT1 knockdown significantly inhibited cell proliferation and migration, which could be reversed by miR-10a-5p inhibitor. Dual-luciferase reporter assay confirmed miR-10a-5p as a common target of NEAT1 and Serine protease inhibitor family E member 1 (SERPINE1). FISH assays revealed the co-localization of NEAT1 and miR-10a-5p in the cytoplasm. Additionally, the methylation level of SERPINE1 in ccRCC was significantly lower than that in normal tissues. Furthermore, SERPINE1 expression was positively correlated with multiple immune cell infiltration levels.

**Conclusions:**

In BAP1-deficient ccRCC, NEAT1 competitively binds to miR-10a-5p, indirectly upregulating SERPINE1 expression to promote kidney cancer cell proliferation. Furthermore, NEAT1/miR-10a-5p/SERPINE1 were found to be independent prognostic factors of ccRCC.

## Introduction

Kidney cancer is a common malignancy, with approximately 430,000 new global cases in 2020 and approximately 170,000 kidney cancer-related deaths (1). The pathology of clear-cell renal cell carcinoma (ccRCC) is characterized by a ‘clear cytoplasm’, owing to its ability to accumulate glycogen and lipids in the cytoplasm. It accounts for up to 80% of all renal cell carcinomas (RCC) and is also considered the most aggressive subtype. Loss or inactivation of tumor suppressors is crucial in tumorigenesis. The role of the classic *VHL* gene and its pathway in ccRCC have been extensively studied (2). Moreover, drugs targeting the VHL–HIF–VEGF pathway, such as sunitinib, sorafenib and axitinib, have been shown to benefit patients with ccRCC, becoming the standard treatment for patients in the advanced stages of ccRCC (3). Unlike other epithelial tumors, mutations in the classic tumor suppressors, such as *BRAF*, *TP53*, *PTEN*, *RB1*, and *EGFR*, are rare in ccRCC (4, 5). In addition to *VHL* inactivation, a recurrent loss of chromosome 3p fragments in ccRCC has been reported (6, 7). Furthermore, BRCA1-Associated Protein 1 (BAP1) on chromosome 3p was identified as a novel tumor-driver gene in ccRCC using extensive parallel sequencing techniques (8, 9).

BAP1 is a novel ubiquitin carboxy-terminal hydrolase and a subfamily member of deubiquitinating enzymes (10, 11). BAP1 is located in the nucleus and binds to *BRCA1* to enhance its tumor suppressor activity (12). Additionally, BAP1 is involved in many biological processes, such as DNA damage repair, cell differentiation and cell proliferation, *via* its deubiquitinating activity (12). Various studies have reported that BAP1 is commonly lost or inactivated in numerous human malignancies, especially in ccRCC, hepatocellular carcinoma and mesothelioma (13). The majority of chromosomes have a 3p deletion, which is an initial marker for nonhereditary ccRCC (14). The mutated frequency of BAP1 was also reported to be as high as 20% in ccRCC (15, 16), with RCC accounting for 9% of BAP1 tumor predisposition syndrome (BAP1-TPDS) (17). Compared with sporadic tumors, BAP1-TPDS RCC has earlier onset, more aggressive tumors and poorer patient survival (17, 18). Due to the poor treatment response, a standard treatment for BAP1 mutated tumors is yet to be identified (19). Recently, several studies have reported the direct targeting of differentially expressed genes in BAP1-deficient tumors (20, 21). For example, histone deacetylase and enhancer of Zeste 2 Polycomb repressive complex 2 are upregulated in BAP1-deficient tumors; therefore, targeting these genes could improve BAP1-deficient tumor sensitivity to treatment (22, 23). Therefore, understanding differentially expressed genes in BAP1-deficient tumors could provide a novel perspective for targeted therapy.

Long noncoding RNAs (lncRNA), longer than 200 nucleotides (nt), are non-protein-coding RNAs that are involved in various tumor developments, including ccRCC, and specific lncRNAs are associated with tumor migration, invasion and poor prognosis (24, 25). MicroRNA (miRNA) is a single-stranded non-coding RNA of approximately 19–25 nt, which can bind to the 3’ untranslated mRNA region and regulate target gene expression (26). Non-coding RNAs (namely, lncRNAs and circular RNAs) could serve as competitive endogenous RNAs (ceRNA) that competitively bind to miRNAs (a post-transcriptional regulator) for cell–cell communication and gene expression co-regulation (27–30).

## Methods and Materials

### Data Collection and Processing

RNAseq (lncRNA, mRNA and miRNA) and relevant clinical data for kidney renal clear cell carcinoma (KIRC) were obtained from the TCGA database (https://portal.gdc.cancer.gov/).

### Identification of Differentially Expressed lncRNAs, miRNAs and mRNAs

Based on the median expression of BAP1, patients were divided into the BAP1^high^ and the BAP1^low^ groups. For differential expression analysis, the cutoff value of DElncRNA was set at |log_2_ FC| >0.5 and *P*. adj <0.05; DEmiRNA with cutoff value of |log_2_ FC| >0.7 and *P*. adj <0.05; DEmRNA with cutoff value of |log_2_ FC| >0.5 and *P*. adj <0.05.

### Construction of ceRNA Networks

Highly conserved microRNA family files were downloaded from the miRcode database (http://mircode.org/). Subsequently, the obtained DElncRNAs were used to find potential miRNAs targeting these DElncRNAs. Then, the selected miRNAs were inputted into the miRBD database (http://mirdb.org/) (31) and the Targetscan database (http://www.targetscan.org/vert_72/) (32) to explore target mRNAs. Finally, the screened DElncRNAs, DEmiRNAs, and DEmRNAs were put into Cytoscape (version 3.6.1) for ceRNA network construction, using the plug-in ‘cytoHubba’ for hub genes network construction (33, 34).

### Functional Annotation

To investigate the functional annotation implicated with DEmRNAs, the Gene Ontology annotation and the Kyoto Encyclopedia of Genes and Genomes pathway analysis were performed using the Metascape website (https://metascape.org/) (35).

### Expression of Hub Genes and Survival Analysis

The expression of the hub genes in tumor and normal tissues based on the ccRCC dataset were compared using the Wilcoxon rank-sum test (*P <*0.05). Overall survival (OS) analysis for the expression of the hub genes between the high- and low-expression groups was performed, with *P <*0.05 indicating statistical significance.

### Clinical Relevance of the Nuclear Enriched Abundant Transcript 1 (NEAT1)/miR-10a-5p/SERPINE1 Axis in Patients With ccRCC

To explore the clinical relevance of the ceRNA axis, the expression levels of NEAT1, miR-10a-5p, and SERPINE1 with different clinical characteristics were evaluated, and the Bonferroni method was used to correct the results of multiple hypothesis testing (Dunn’s test, *P*. adj <0.05). Moreover, univariate and multivariate Cox regression analyses were conducted to investigate the prognostic significance of clinical features.

### Cell Culture

The ACHN and 786-O cell lines were obtained from the American Type Culture Collection (Manassas, VA, USA). Both cell lines were cultured in Dulbecco’s modified Eagle’s medium (Gibco) containing 10% fetal bovine serum (Gibco) and 1% penicillin/streptomycin and incubated at 37°C in a humidified 5% CO_2_ atmosphere.

### Cell Transfection

Cells were seeded into six-well plates and cultured until the cell density reached approximately 60%. The cells were then transfected with small-interfering RNA (siRNA-NEAT1#1, #2) or miR-10a-5p mimic/inhibitor, using jetPRIME^®^ transfection reagent (NY, USA) following the protocol of the manufacturer. Additionally, a relevant negative control (NC) was used. These siRNAs, miR-10a-5p mimic/inhibitor and relevant NC were designed by GenePharma (Shanghai, China).

### RT-qPCR (Quantitative Reverse Transcription-Polymerase Chain Reaction) and Western Blotting

Total RNA was extracted using RNAeasy™ (Beyotime; Shanghai, China), following the instructions of the manufacturer. HiScript^®^ II reverse transcriptase (Vazyme; Nanjing, China) was used to convert RNA to cDNA. RT-qPCR was performed in a LineGene 9600 Plus system (Bioer Technology, Hangzhou, China), using 2 × SYBR Green qPCR Master Mix (High ROX; Servicebio; Wuhan, China), following the protocol of the manufacturer. The expression of miR-10a-5p was normalized to control U6, while that of the others were normalized to GAPDH, and relative expression was calculated using the 2^−ΔΔCt^ method. Western blotting was performed as previously described (36), and anti-SERPINE1 (Rabbit Polyclonal Antibody, AF7965, Beyotime), anti-GAPDH (Mouse Monoclonal Antibody, AF5009, Beyotime) antibodies were used for further experimentation.

### Cell Viability

After 48 h of transfection, 100 µl cell suspension containing 1 × 10^3^ cells were seeded into 96-well plates and cultured. After 24, 48, 72, and 96 h, 10 µl of CCK-8 (Beyotime; Shanghai, China) was added to each well and incubated at 37°C for another 2 h. The absorbance was detected using a microplate reader at 450 nm.

### Colony Formation Assay

After transfection for 48 h, the cell suspension was added to six-well plates with approximately 1 × 10^3^ cells per well and incubated at 37°C in a humidified 5% CO_2_ atmosphere for 2 weeks. After culturing, the cells were washed twice with PBS, fixed with 4% paraformaldehyde for 15 min, stained with 0.1% crystal violet for 15 min, washed thrice with PBS again. The number of cell colonies with a diameter of >0.1 mm was further observed under the microscope.

### Wound Healing Assay

The treated cells were seeded into six-well plates. When the cell density was close to 90%, a linear scratch was made using a 200 µl plastic pipette tip. The wound-healing time in different treatment groups were observed under a microscope and photographed at 0 and 12 h. The wound closure rate was measured thrice and averaged.

### Luciferase Reporter Assays

The dual-luciferase reporter gene plasmids containing wild-type and mutant sequences were synthesized using Promega (Madison, WI, USA). The wild-type or mutant (NEAT1, SERPINE1) reporter plasmid and miR-10a-5p mimic or mimic-NC were co-transfected into 293T cells. Luciferase activity was measured 48 h after transfection.

### Fluorescence *In Situ* Hybridization (FISH)

FISH analysis on the ACHN cells was performed to determine the subcellular localization of *NEAT1* and miR-10a-5p. The RNA probes were designed and synthesized *via* Servicebio (Wuhan, China). Briefly, the cells were fixed with 4% paraformaldehyde for 20 min and washed with PBS. Then, proteinase K (20 ug/ml) was digested for 8 min, followed by PBS washes. Subsequently, a pre-hybridization solution was added for 1 h at 37°C and then discarded. RNA probes containing a hybridization solution was added to the cells and hybridized overnight at 37°C. Next, DAPI staining solution was added after the washes and incubated for 8 min, followed by rinsing and the addition of an anti-fluorescence quenching blocker dropwise to seal the slice. Finally, the slices were visualized under a Nikon fluorescence microscope.

### DNA Methylation Analysis of SERPINE1

To investigate the DNA methylation level of SERPINE1, co-expression analysis of SEPRINE1 was conducted using three DNA methyltransferases (DNMT1, DNMT3A, DNMT3B). Following this, methylation analysis of SERPINE1 was performed between tumor and normal tissues using the online database DiseaseMeth version 2.0 (http://bio-bigdata.hrbmu.edu.cn/diseasemeth/). MEXPRESS (https://mexpress.be/) was used to further determine the relationship between *SERPINE1* expression and DNA methylation status (37).

### Correlation Between Immune Infiltration and Expression of SERPINE1 in KIRC

TIMER (https://cistrome.shinyapps.io/timer/) was used for the comprehensive analysis of the relationship between SERPINE1 expression and tumor-infiltrating immune cell levels, namely, neutrophils, macrophages, dendritic cells, B cells, CD4^+^ T cells, and CD8^+^ T cells (38).

### Software and Versions

R software (x64, version 4.0.3) was used for the statistical analyses (https://www.r-project.org/).

## Results

### BAP1 Acts as a Tumor Suppressor in ccRCC

**Figure 1:**
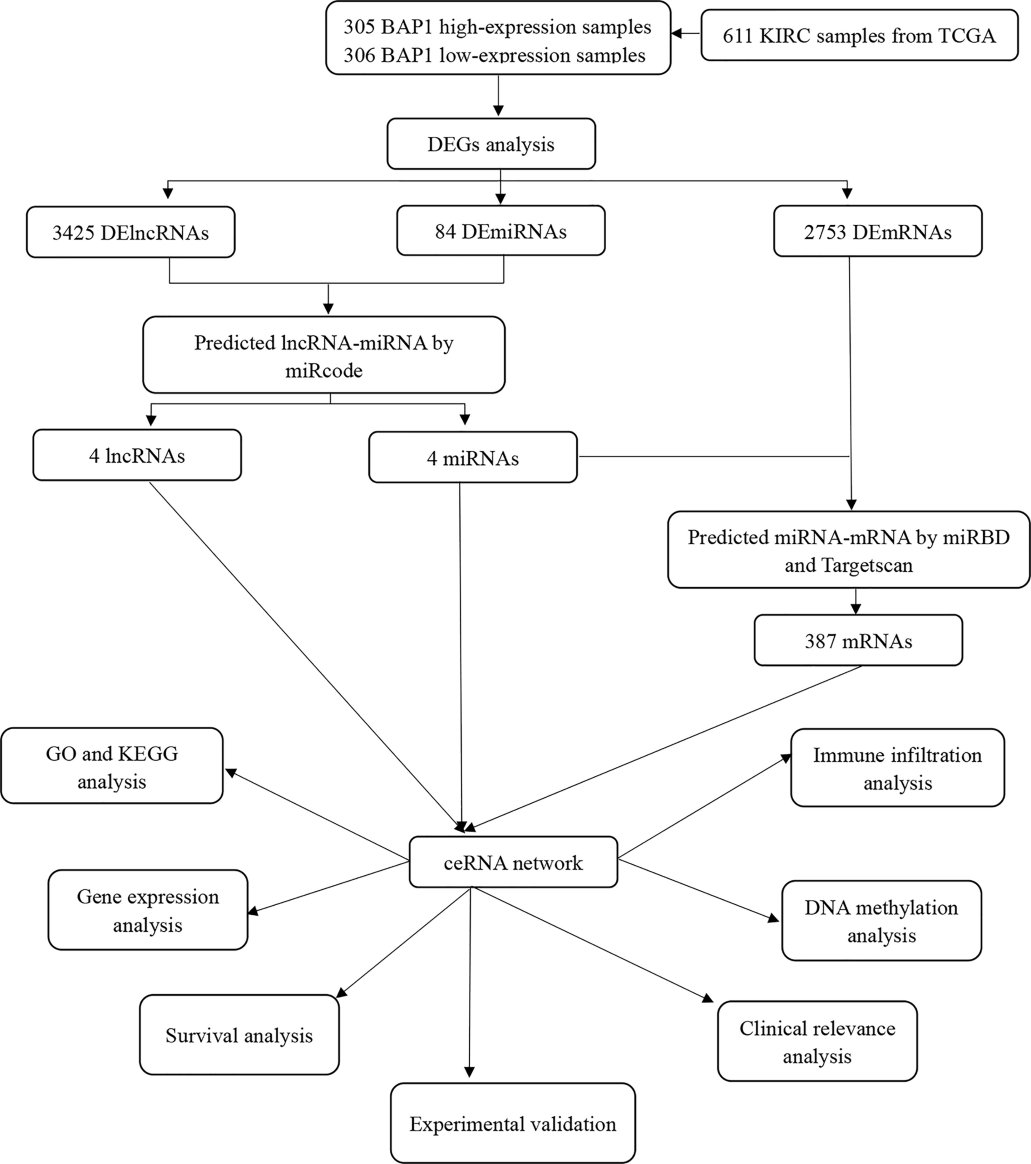
Flow diagramm of ceRNA construction and analysis.

**Figure 2:**
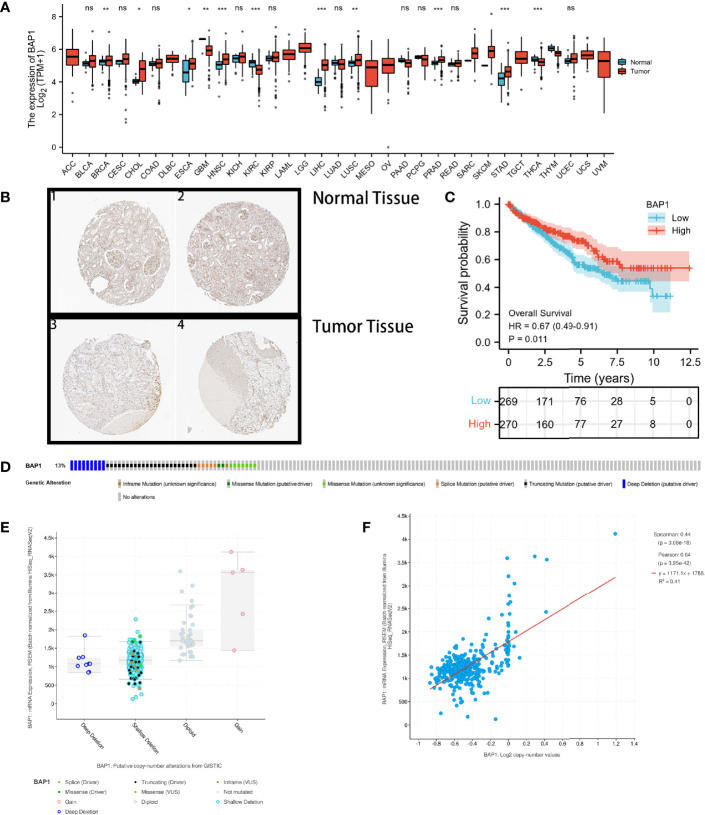
BAP1 acts as a tumor suppressor in kidney renal clear cell carcinoma (KIRC). **(A)** Pan-cancer analysis of BAP1; **(B)** Immunohistochemical analysis of BAP1 in renal tumor and normal tissues; **(C)** Survival analysis comparing high- and low expression of BAP1; **(D)** Distribution of BAP1 genomic alterations inTCGA KIRC; **(E, F)** Relationship between copy number alterations and BAP1 expression: scatter plot **(E)**, correlation plot **(F)**. ns, not significant, p ≥ 0.05; *p < 0.05; **p < 0.01; ***p < 0.001.

**Figure 1** presents a flow diagram of ceRNA construction and analysis. A pan-cancer analysis was performed to evaluate the RNA and protein expression level of BAP1 using data from the UCSC XENA (https://xenabrowser.net/datapages/) and the Human Protein Atlas (HPA) databases (https://www.proteinatlas.org/), respectively. BAP1 RNA expression was found to be significantly downregulated in ccRCC (**Figure 2A**), while BAP1 protein level was the lowest in renal cancer (**Figure S1**). Furthermore, immunohistochemistry staining data obtained from the HPA validated BAP1 downregulation in tumor tissue (**Figure 2B** and **Table S1**). Additionally, Kaplan–Meier survival curves indicated that the low expression of BAP1 was associated with poor OS in ccRCC (**Figure 2C**).

Moreover, the cBioPortal (http://www.cbioportal.org/) was used to explore the potential mechanisms underlying the abnormally low expression of BAP1 in ccRCC (39). **Figure 2D** shows that the genetic alteration rate of BAP1 was found to be 13%, with gene deletions (deep deletion and shallow deletion) accounting for more than half of the copy number alterations in ccRCC samples (**Figure 2E**). Additionally, the mRNA expression level of BAP1 was found to be positively correlated with the copy number value (**Figure 2F**).

### Identification of DERNAs and lncRNA–miRNA–mRNA Networks

**Figure 3:**
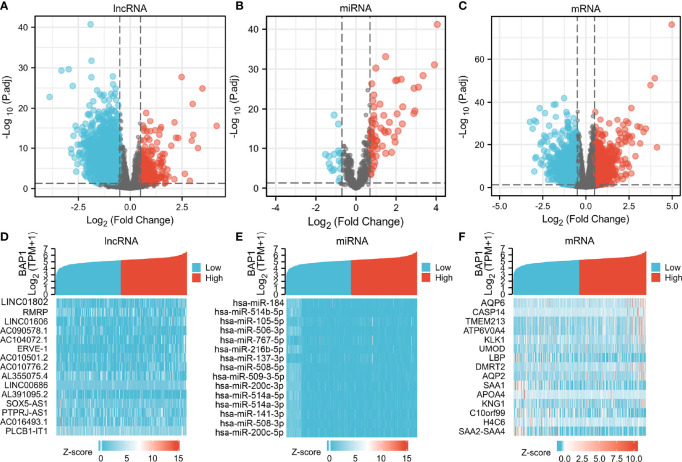
Volcano plots and heatmap plots of DElncRNAs, DEmiRNAs, and DEmRNAs between the expression of BAP1high and BAP1low in KIRC samples. Red color represents up-regulated genes, blue represents down-regulated genes. **(A)** 3425 DElncRNAs (|log2 FC| > 0.5 and P. adj < 0.05); **(B)** 84 DEmiRNAs (|log2 FC| > 0.7 and P. adj < 0.05); **(C)** 2753 DEmRNAs with cutoff value of |log2 FC| > 0.5 and P. adj < 0.05; **(D–F)** Heatmaps of the top 15 significant DElncRNAs, miRNAs and mRNAs.

**Figure 4:**
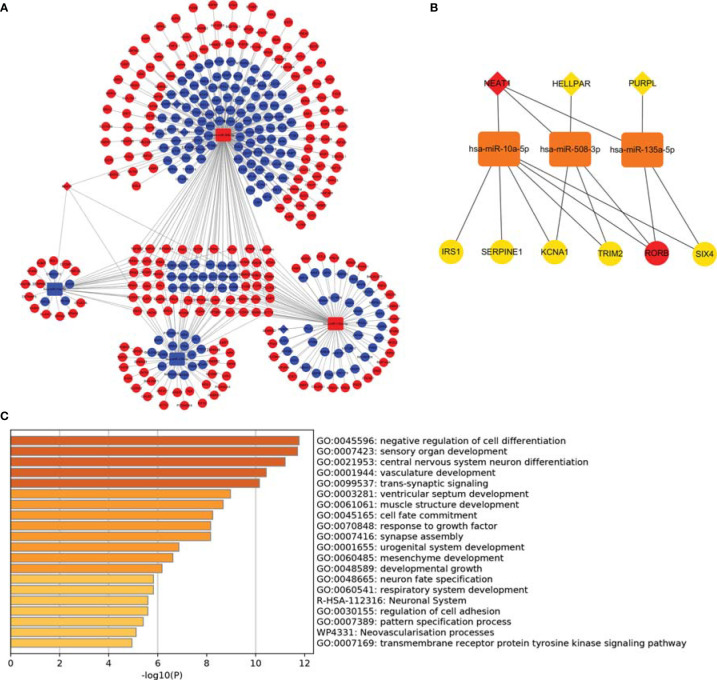
Construction of ceRNA networks and functional annotation. **(A)** A triple regulatory network based on 4 lncRNAs, 4 miRNAs, and 387 mRNAs; **(B)** Hub genes network was constructed using plug-in “cytoHubba”; **(C)** Functional enrichment analysis of DEmRNAs.

DERNAs were screened according to the cut-off value (**Figure 3**) and intersected with the predicted RNAs. The shortlisted genes were inputted into Cytoscape for hub genes network construction (**Figure 4A**). Finally, lncRNAs (NEAT1, HELLPAR, PURPL), miRNAs (miR-10a-5p, miR-508-3p, miR-135a-5p) and mRNAs (IRS1, SERPINE1, KCAN1, TRIM2, RORB, SIX4) were identified (**Figure 4B**). A functional enrichment analysis of DEmRNAs demonstrated their involvement in mesenchyme development, transmembrane receptor protein tyrosine kinase signaling pathway and cell adhesion regulation (**Figure 4C**).

### Construction of Prognostic-Related ceRNA in ccRCC

**Figure 5:**
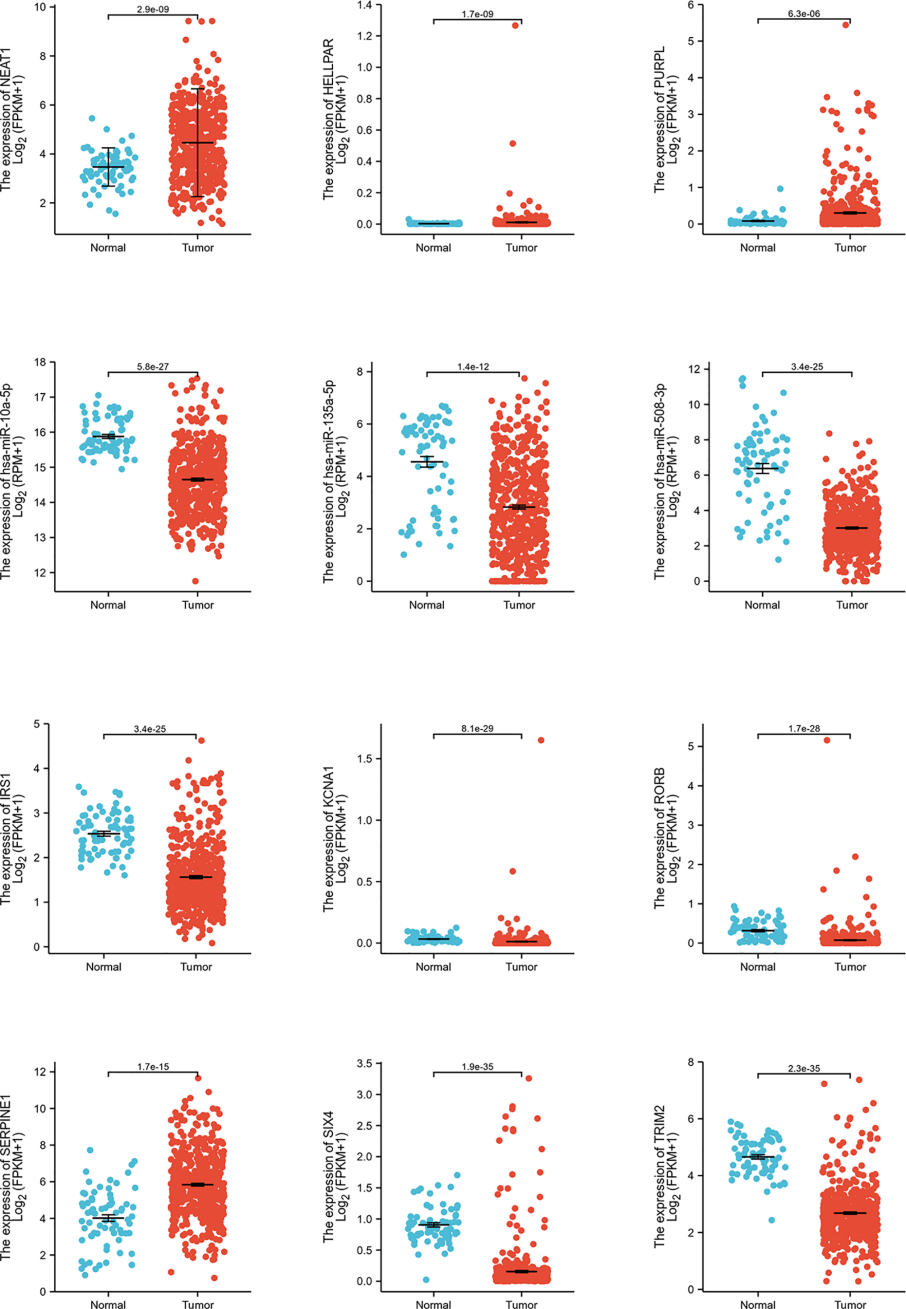
Hub genes expression analysis. Expression analysis of 12 hub genes (3 lncRNAs, 3 miRNAs, 6 mRNAs) comparing tumor and normal tissues.

**Figure 6:**
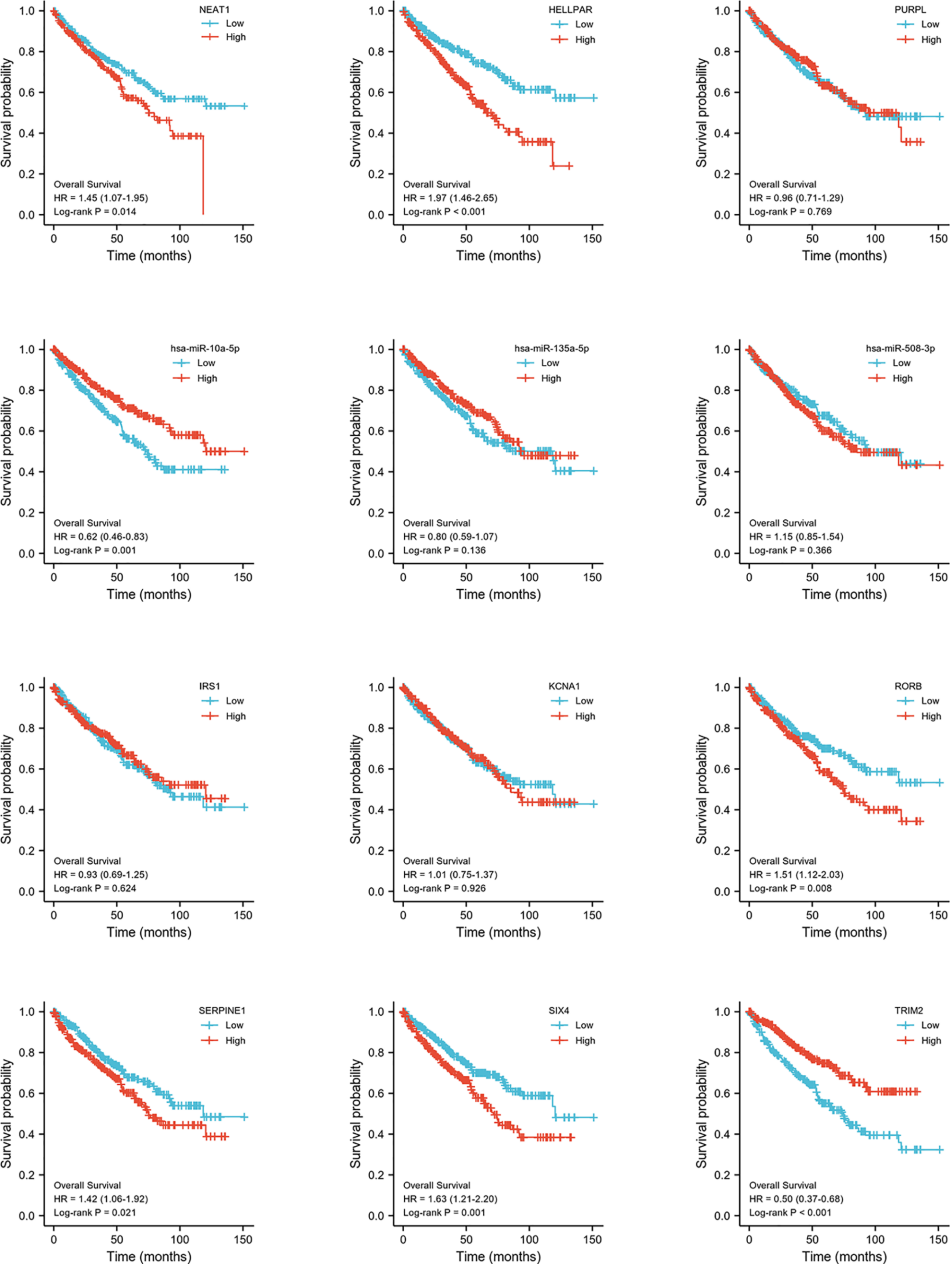
Hub genes survival analysis. Survival analysis of 12 hub genes (3 lncRNAs, 3 miRNAs, 6 mRNAs) comparing high- and low expression group.

**Figure 7:**
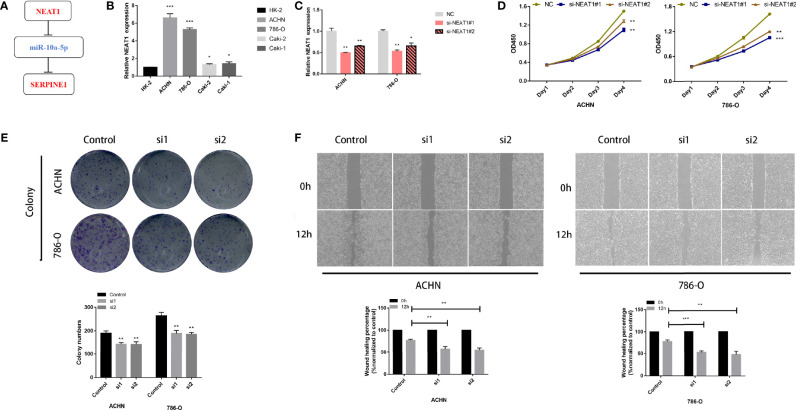
LncRNA NEAT1 promotes proliferation and migration in kidney cancer cell lines. **(A)** Construction of a ceRNA axis; **(B)** NEAT1 is upregulated in kidney cancer cell lines; **(C)** Validation of knockdown efficiency of siNEAT1 by RT-qPCR; Knockdown of NEAT1 inhibits proliferation **(D)**, colony formation **(E)** and migration **(F)** of kidney cancer cells. p≥0.05; *p < 0.05; **p < 0.01; ***p < 0.001.

Expression and survival analyses of hub genes are shown in **Figures 5**, **6**. In total, two DElncRNAs (NEAT1, HELLPAR), one DEmiRNA (miR-10a-5p) and four DEmRNAs (SERPINE1, TRIM2, RORB, SIX4) were found to be prognostic-related genes. Moreover, the lncLocator (www.csbio.sjtu.edu.cn/bioinf/lncLocator/) showed that NEAT1 was mainly distributed in the cytoplasm (**Figure S2**), indicating its role as a ceRNA in enhancing SERPINE1 expression by sponging miR-10a-5p. Finally, a prognostic-related NEAT1/miR-10a-5p/SERPINE1 ceRNA network was constructed (**Figure 7A**).

### Clinical Relevance of the NEAT1/miR-10a-5p/SERPINE1 Axis in Patients With ccRCC

In this study, NEAT1 was correlated with gender (*P <*0.05, **Figure S3A**). The lower expression level of miR-10a-5p was associated with higher T stage, M stage, pTNM stage, tumor grade and gender (*P <*0.05, **Figure S3B**). Moreover, SERPINE1 was strongly correlated with T stage, N stage, pTNM stage, tumor grade and gender (*P <*0.05, **Figure S3C**). The multivariate Cox regression analysis showed that NEAT1 (hazard ratio (HR) = 1.488, *P* = 0.011), SERPINE1 (HR = 1.456, *P* = 0.015) and miR-10a-5p (HR = 0.681, *P =* 0.014) were independent prognostic factors in ccRCC (**Tables S2–4**). The AUC (area under the curve) of the receiver operating characteristics (ROC) analysis (**Figure S4**) indicated a good prognostic performance of SERPINE1 (AUC = 0.789) and miR-10a-5p (AUC = 0.892). Additionally, the pan-cancer analysis showed that SERPINE1 mRNA was highly expressed in kidney cancer (**Figure S5A**). Furthermore, immunohistochemical analysis revealed that SERPINE1 was located in the cytoplasmic/membranous area (**Figure S5B**).

### lncRNA NEAT1 Regulates Tumor Proliferation and Migration in Kidney Cancer Cell Lines

RT-qPCR analysis showed that NEAT1 was highly expressed in kidney cancer cell lines compared to that in HK-2, with more significant differences in 786-O and ACHN cell lines (**Figure 7B**). The knockdown efficiency of the two siRNAs was verified using RT-qPCR (**Figure 7C**). CCK-8 and colony formation assays showed that 786-O and ACHN cell proliferation was significantly suppressed after NEAT1 knockdown (**Figures 7D, E**). Wound healing assays showed that the migration ability of the cells was inhibited after transfection with siNEAT1 (**Figure 7F**).

### NEAT1 Serves as a Sponge for miR-10a-5p and Upregulates SERPINE1 to Regulate the Proliferation of Kidney Cancer Cells

**Figure 8:**
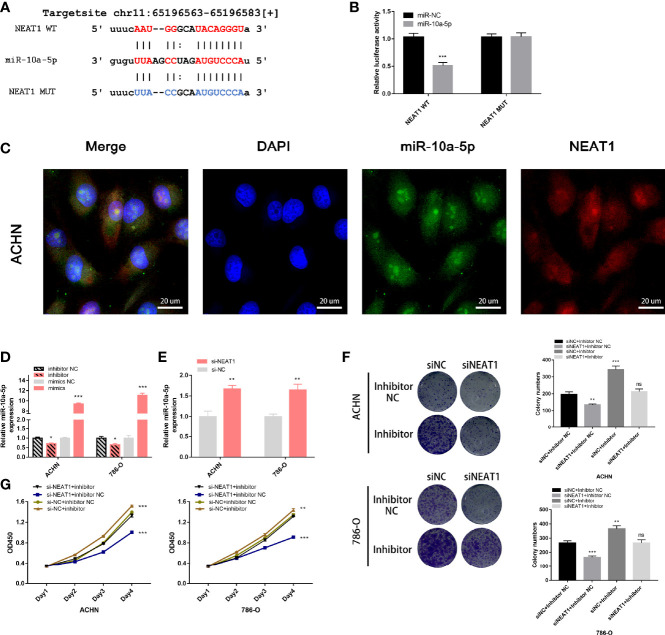
NEAT1 serves as sponge for miR‑10a-5p in kidney cancer cells. **(A)** Prediction the target sequence of NEAT1 bonding to miR-10a-5p by starBase; **(B)** Dual luciferase reporter gene assays verify the direct binding of miR-10a-5p to NEAT1 target sequence; **(C)** FISH assays confirm that NEAT1 co-localizes with miR-10a-5p in the cytoplasm of kidney cancer cells; **(D)** Validation the efficiency of miR-10a-5p mimic and inhibitor by RT-qPCR; **(E)** Knockdown of NEAT1 enhances the expression of miR-10a-5p; **(F, G)** miR-10a-5p inhibitor reverses the inhibition of cell proliferation caused by siNEAT1. ns, not significant, p ≥ 0.05; *p < 0.05; **p < 0.01; ***p < 0.001.

The target site of NEAT1 to miR-10a-5p was predicted using the starBase (https://starbase.sysu.edu.cn/), and the wild-type and mutant sequences are shown in **Figure 8A**. A dual fluorescein reporter gene plasmid (NEAT1-WT/NEAT1-MUT) was constructed and co-transfected into 293T cells with miR-10a-5p mimic and miR-NC. Overexpression of miR-10a-5p significantly reduced luciferase activity in the NEAT1-WT group but not in the NEAT1-MUT group (**Figure 8B**), confirming that miR-10a-5p binds directly to NEAT1. Moreover, FISH assays revealed that miR-10a-5p co-localized with NEAT1 in the cytoplasm of ACHN cells (**Figure 8C**). Therefore, NEAT1 serves as a sponge for miR-10a-5p and inhibit its function in kidney cancer cells.

Next, the efficiency of the miR-10a-5p mimic and inhibitor on miR-10a-p expression was verified using RT-qPCR (**Figure 8D**). On transfection with siNEAT1 in ACHN and 786-O cells, miR-10a-5p expression was significantly enhanced (**Figure 8E**). Therefore, co-transfection siRNA and miR-inhibitor (siNC + inhibitor NC; siNC + inhibitor; siNEAT1 + inhibitor NC; siNEAT1 + inhibitor) in ACHN and 786-O cells, CCK-8 and colony formation assays suggested that the knockdown of NEAT1 on the suppression of cell proliferation and colony formation in ACHN and 786-O cells could be reversed by a miR-10a-5p inhibitor (**Figures 8F, G**).

**Figure 9:**
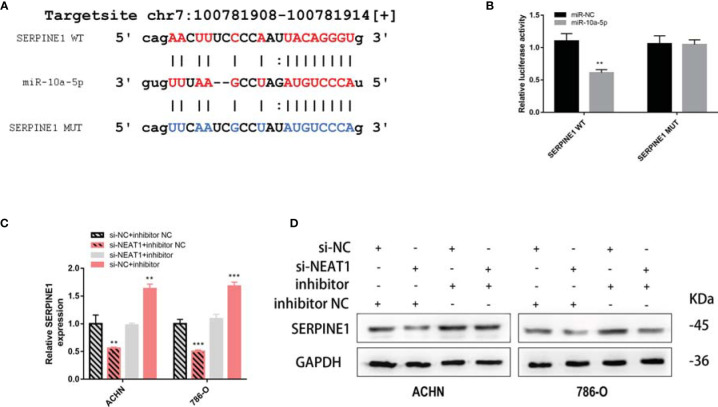
NEAT1 sponging miR-10a-5p to regulate SERPINE1. **(A)** Prediction the target sequence of SERPINE1 bonding to miR-10a-5p by starBase; **(B)** Dual luciferase reporter gene assays verify the direct binding of miR-10a-5p to SERPINE1 target sequence; **(C, D)** Knockdown of NEAT1 can downregulate the expression of SERPINE1 but can be reversed by miR-10a-5p inhibitor. **p < 0.01; ***p < 0.001.

To confirm that miR-10a-5p regulates the expression of SERPINE1 by binding directly to the target sequence, a luciferase reporter gene plasmid was constructed for SERPINE1-WT/MUT (**Figure 9A**). The results showed that miR-10a-5p mimic significantly reduced the luciferase activity of SERPINE1-WT; however, no significant changes were observed for SERPINE1-MUT (**Figure 9B**). siNEAT1 was co-transfected with miR-10a-5p inhibitor into ACHN and 786-O cells and evaluated using RT-qPCR and western blot to verify the regulation of SERPINE1 expression. The results demonstrated that the knockdown of NEAT1 significantly downregulated the expression of SERPINE1; however, this effect could be reversed by a miR-10a-5p inhibitor (**Figures 9C, D**).

### DNA Methylation Analysis of SERPINE1

**Figure 10:**
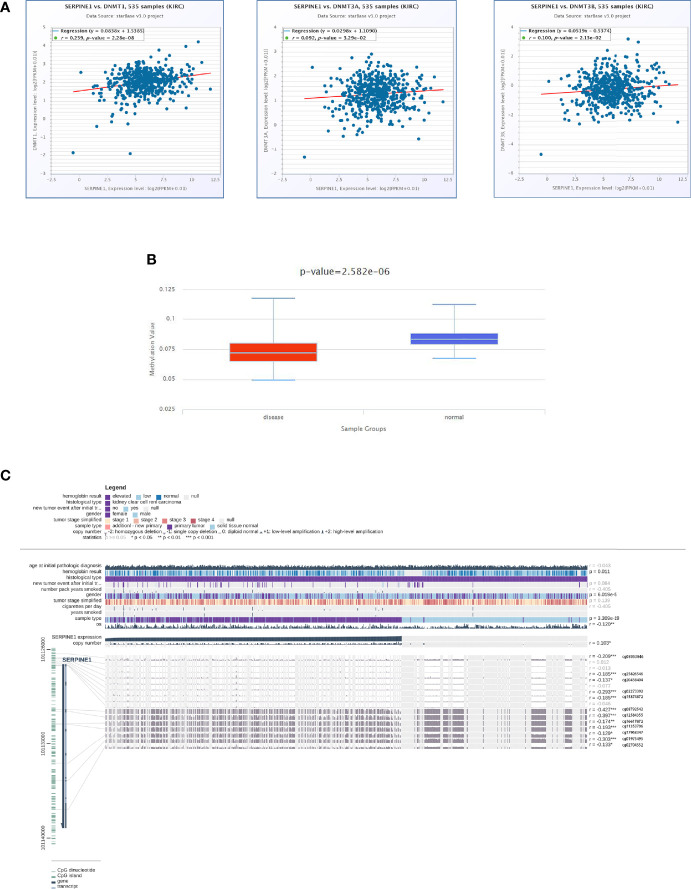
DNA methylation analysis of SERPINE1. **(A)** Co-expression analysis of SERPINE1 with three DNMTS (DNMT1, DNMT3A and DNMT3B); **(B)** Methylation level of SERPINE1 comparing KIRC and normal samples; **(C)** Relationship between SERPINE1 expression and genome-wide methylation by MEXPRESS.

To elucidate the mechanism of the abnormally high expression of SERPINE1 in ccRCC, a series of methylation analyses of SERPINE1 was performed. Co-expression analysis suggested that SERPINE1 expression was positively correlated with DNMT1, DNMT3A, and DNMT3B expression levels (*P <*0.05, **Figure 10A**). Additionally, the methylation level of SERPINE1 in normal tissues was much higher than ccRCC tissue samples (*P <*0.001, **Figure 10B**). Moreover, 12 DNA methylation sites that were negatively correlated with SERPINE1 expression were identified (**Figure 10C**).

### Immune Infiltration Analysis of SERPINE1 in KIRC

**Figure 11:**
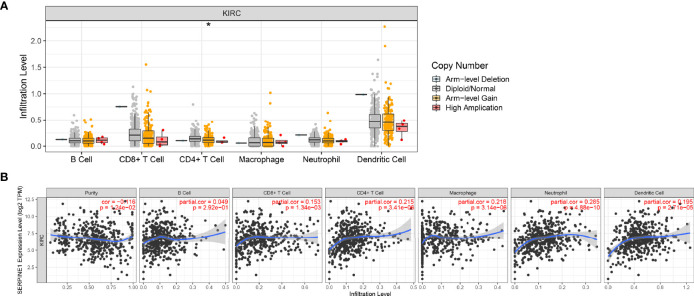
Immune infiltration analysis of SERPINE1 in KIRC. **(A)** Relationship between the level of immune cell infiltration and the copy number of SERPINE1 in KIRC. **(B)** Correlation of SERPINE1 expression and immune cell infiltration in KIRC. *p < 0.05.

To further investigate the relationship between SERPINE1 expression and the immune microenvironment in ccRCC, an immune infiltration analysis was performed using TIMER. ‘SCNA’ module analysis indicated that the immune infiltration level of CD 4^+^ T cell was associated with the altered copy numbers of SERPINE1 (**Figure 11A**). Moreover, ‘Gene’ module analysis showed that the expression of SERPINE1 was positively related to the immune infiltration level of CD4^+^ T cell, CD8^+^ T cell, macrophages, dendritic cells and neutrophils (**Figure 11B**).

## Discussion

Numerous studies have shown that transcription occurs in approximately 80% of the human genome, with protein-coding genes accounting for only 2% of the human genome. This suggests that the majority of RNAs are non-coding genes (40). Cancer is often associated with abnormal transcriptomes; moreover, increasing evidence indicates that the non-coding transcriptome is often dysregulated in cancer and plays an important role in determining its pathogenesis (41, 42). Therefore, on analyzing the differentially expressed non-coding RNAs in BAP1-deficient ccRCC, this study identified NEAT1/miR-10a-5p/SERPINE1 as a BAP1-related prognostic ceRNA.

NEAT1, which is located in the nuclear paraspeckles, has been reported to be involved in various biological processes, such as tumorigenesis, infection, neuropathy, and immunity (43–46). Previous studies have shown that NEAT1 plays a key role in carcinogenesis by mediating gene expression. For example, NEAT1 induces transcription factors to relocate from the promoters of downstream genes to the paraspeckles, altering gene transcription (44). NEAT1 also acts as a scaffold to bind multiple proteins located in the paraspeckles together or as a bridge for protein complexes (47, 48). Recently, NEAT1 has been reported to act as a ceRNA to regulate the expression of downstream genes by sponging miRNAs in malignant tumors (49–51). Previous studies have demonstrated that BRCA1 as a transcription factor can directly bind 1.4 kb upstream of the TSS region of NEAT1 and negatively regulate its expression (52). In the nucleus, BAP1 binds to BRCA1 and enhances its tumor suppressive activity. Thereby, the loss of BAP1 was hypothesized to indirectly affect the tumor suppressor activity of BRCA1, resulting in an abnormally high expression of NEAT1.

SERPINE1, encoding plasminogen activator inhibitor 1 (PAI-1), is an essential inhibitor of tissue plasminogen activator and urokinase (uPA). Previous studies have focused on the effect of SERPINE1 on human thrombosis, cardiac fibrosis, inflammatory injury, ageing and metabolism (53–57). However, current studies report on the importance of SERPINE1 in promoting tumor malignant progression, distant metastasis and chemotherapy resistance through multiple pathways. Moreover, high SERPINE1 expression is significantly associated with poor prognosis (58, 59). The underlying mechanism has been speculated to be the migratory effect of uPA–uPAR–PAI-1 systems on endothelial cells, with fibrin deposition playing an important role in tumor angiogenesis (60); SERPINE1 also functions as an extracellular matrix (ECM) component to stabilize tumor cell adhesion in migration (61). ECM is composed of proteins and proteoglycans that are secreted by keratinocytes, fibroblasts and immune cells (62). In the complex tumor microenvironment (TME), dynamic cell–cell and cell–ECM interactions play a crucial role in tumor initiation and immune cell regulation (63). The tumor immune microenvironment determines the biological behaviour of tumour cells, with immune cell infiltration levels correlating with tumor prognosis (64–66). In this study, the copy number of SERPINE1 was found to be associated with the immune infiltration of CD4^+^ T cells, and the expression of SERPINE1 was related to the level of immune infiltration of CD4^+^ T cells, CD8^+^ T cells, macrophages, dendritic cells and neutrophils. Roelofs et al. demonstrated that SERPINE1 regulated neutrophil influx during renal fibrosis, suggesting that SERPINE1 acts as a chemokine to mediate immune cell infiltration (67). Moreover, in oral squamous cell carcinoma, PAI-1 has been shown to induce CD14^+^ monocytes to differentiate into CD206^+^ tumor-associated macrophages (TAMs), producing epidermal growth factors to mediate tumor cell migration (68). In the TME, cancer-associated fibroblasts induce the M2-polarization of macrophages and produce chemokine ligand 12 to promote the secretion of PAI-1 in TAMs, leading to the malignant process of hepatocellular carcinoma (69).

Current anti-cancer drug research uses a two-dimensional model of cytotoxicity *in vitro* (63), which does not accurately represent the three-dimensional TME. Moreover, the individual differences and intra-tumor cell heterogeneity are not sufficiently considered, resulting in many clinically ineffective anti-cancer drugs (70). Therefore, targeting the abnormally elevated functional proteins of ECMs in the TME could be a new direction for the development of anti-tumor drugs. In this study, the loss of BAP1 resulted in an abnormal upregulation of SERPINE1 (PAI-1) and hence, SERPINE1 could be a new target for the treatment of BAP1-deficient ccRCC.

## Conclusions

A ceRNA (NEAT1/miR-10a-5p/SERPINE1) network was constructed that could be used as a prognostic biomarker of BAP1-deficient ccRCC. Furthermore, miR-10a-5p/SERPINE1 was significantly associated with clinical features, indicating their role as independent prognostic factors of ccRCC.

## Data Availability Statement

The original contributions presented in the study are included in the article/**Supplementary Material**. Further inquiries can be directed to the corresponding authors.

## Author Contributions

RJL designed the study and conducted data extraction, analysis and experimentation. RJL, ZPX, SYL, JJY, and BX wrote the manuscript. RJL, ZPX, NHF, BX, and MC reviewed and revised the manuscript. All authors listed have made a substantial, direct, and intellectual contribution to the work and approved it for publication.

## Funding

This study was funded by The National Natural Science Foundation of China (Nos. 81872089, 81370849, 81672551, 81300472, 81070592, 81202268, 81202034); the Six Talent Peaks Project in Jiangsu Province, Jiangsu Provincial Medical Innovation Team (CXTDA2017025); the Natural Science Foundation of Jiangsu Province (BK20161434, BL2013032, BK20150642, and BK2012336); the Major Project of Jiangsu Commission of Health: (No: ZD2021002); the Wuxi ‘Taihu Talents Program’ Medical Expert Team Project (Nos. THRCJH20200901, THRCJH20200902).

## Conflict of Interest

The authors declare that the research was conducted in the absence of any commercial or financial relationships that could be construed as a potential conflict of interest.

## Publisher’s Note

All claims expressed in this article are solely those of the authors and do not necessarily represent those of their affiliated organizations, or those of the publisher, the editors and the reviewers. Any product that may be evaluated in this article, or claim that may be made by its manufacturer, is not guaranteed or endorsed by the publisher.
