# Supplementary material for: BAP1-Related ceRNA (NEAT1/miR-10a-5p/SERPINE1) Promotes Proliferation and Migration of Kidney Cancer Cells
Source: Front Oncol. 2022 Mar 29;12:852515. doi: 10.3389/fonc.2022.852515 (PMC9004599; doi:10.3389/fonc.2022.852515)
Supplement: Supplementary file 1 [file DataSheet_1.docx]

**Table. S1. Detailed immunohistochemical information from HPA database.**

| Factor | Total (N) | Univariate analysis | | Multivariate analysis | |
| --- | --- | --- | --- | --- | --- |
|  |  | HR (95%CI) | *P* value | HR (95%CI) | *P* value |
| NEAT1 (High vs. Low) | 539 | 1.429 (1.056-1.934) | 0.021 | 1.488 (1.097-2.020) | 0.011 |
| Pathologic stage (Stage II & Stage III & Stage IV vs. Stage I) | 536 | 3.299 (2.342-4.648) | <0.001 | 3.270 (2.321-4.607) | <0.001 |
| Age (>60 vs. <=60) | 539 | 1.765 (1.298-2.398) | <0.001 | 1.733 (1.273-2.359) | <0.001 |
| Histologic grade (G2 & G3 & G4 vs. G1) | 531 | 9231340.001 (0.000-Inf) | 0.992 |  |  |
| Gender (Male vs. Female) | 539 | 0.930 (0.682-1.268) | 0.648 |  |  |

**Table. S2. Univariate and Multivariate Cox regression analysis of NEAT1 in ccRCC patients.**

**Table. S3. Univariate and Multivariate Cox regression analysis of miR-10a-5p in ccRCC patients.**

| Factor | Total (N) | Univariate analysis | | Multivariate analysis | |
| --- | --- | --- | --- | --- | --- |
|  |  | HR (95%CI) | *P* value | HR (95%CI) | *P* value |
| miR-10a-5p (High vs. Low) | 545 | 0.613 (0.453-0.830) | 0.002 | 0.681 (0.502-0.925) | 0.014 |
| Pathologic stage (Stage II & Stage III & Stage IV vs. Stage I) | 542 | 3.192 (2.272-4.484) | <0.001 | 2.994 (2.128-4.211) | <0.001 |
| Age (>60 vs. <=60) | 545 | 1.849 (1.361-2.512) | <0.001 | 1.737 (1.277-2.362) | <0.001 |
| Histologic grade (G2 & G3 & G4 vs. G1) | 535 | 9267870.436 (0.000-Inf) | 0.991 |  |  |
| Gender (Male vs. Female) | 545 | 0.918 (0.674-1.251) | 0.588 |  |  |

**Table. S4. Univariate and Multivariate Cox regression analysis of SERPINE1 in ccRCC patients.**

| Factor | Total (N) | Univariate analysis | | Multivariate analysis | |
| --- | --- | --- | --- | --- | --- |
|  |  | HR (95%CI) | *P* value | HR (95%CI) | *P* value |
| SERPINE1 (High vs. Low) | 539 | 1.424 (1.054-1.923) | 0.021 | 1.456 (1.075-1.972) | 0.015 |
| Pathologic stage (Stage II & Stage III & Stage IV vs. Stage I) | 536 | 3.299 (2.342-4.648) | <0.001 | 3.200 (2.271-4.509) | <0.001 |
| Age (>60 vs. <=60) | 539 | 1.765 (1.298-2.398) | <0.001 | 1.728 (1.269-2.352) | <0.001 |
| Histologic grade (G2 & G3 & G4 vs. G1) | 531 | 9231340.001 (0.000-Inf) | 0.992 |  |  |
| Gender (Male vs. Female) | 539 | 0.930 (0.682-1.268) | 0.648 |  |  |


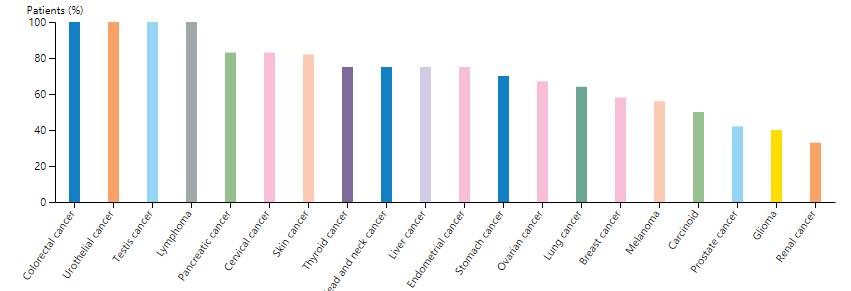


**Figure S1. BAP1 protein level in pan-cancer.**

**
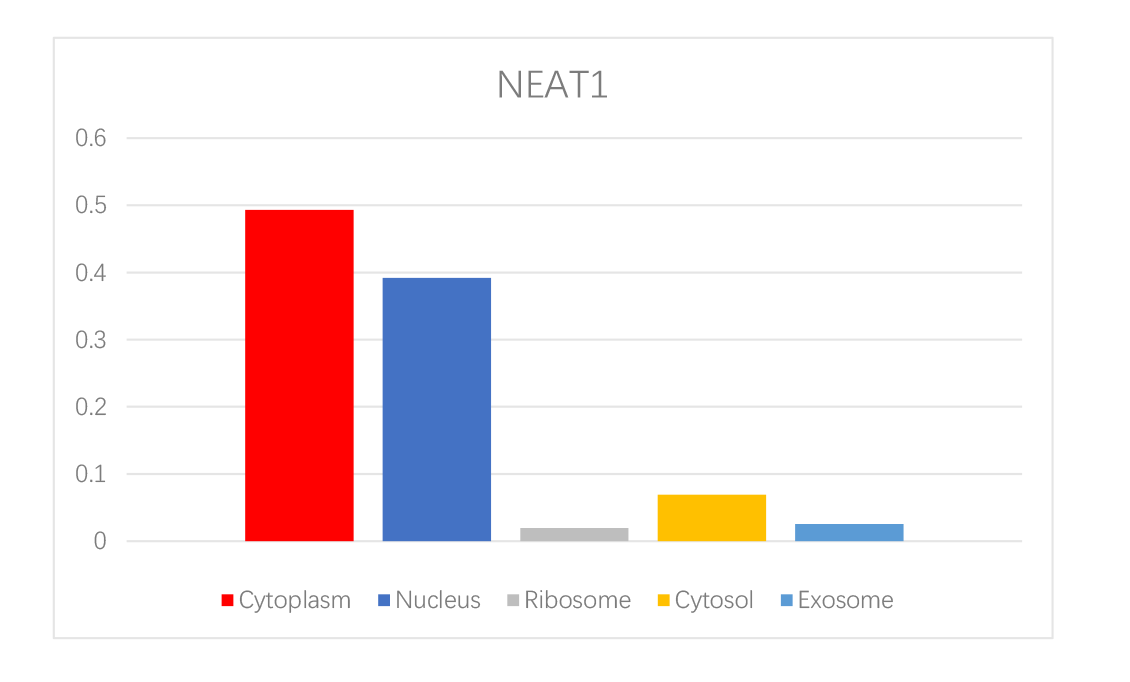
**

**Figure S2.** **Subcellular localization analysis of NEAT1 with lncLocator.**

**
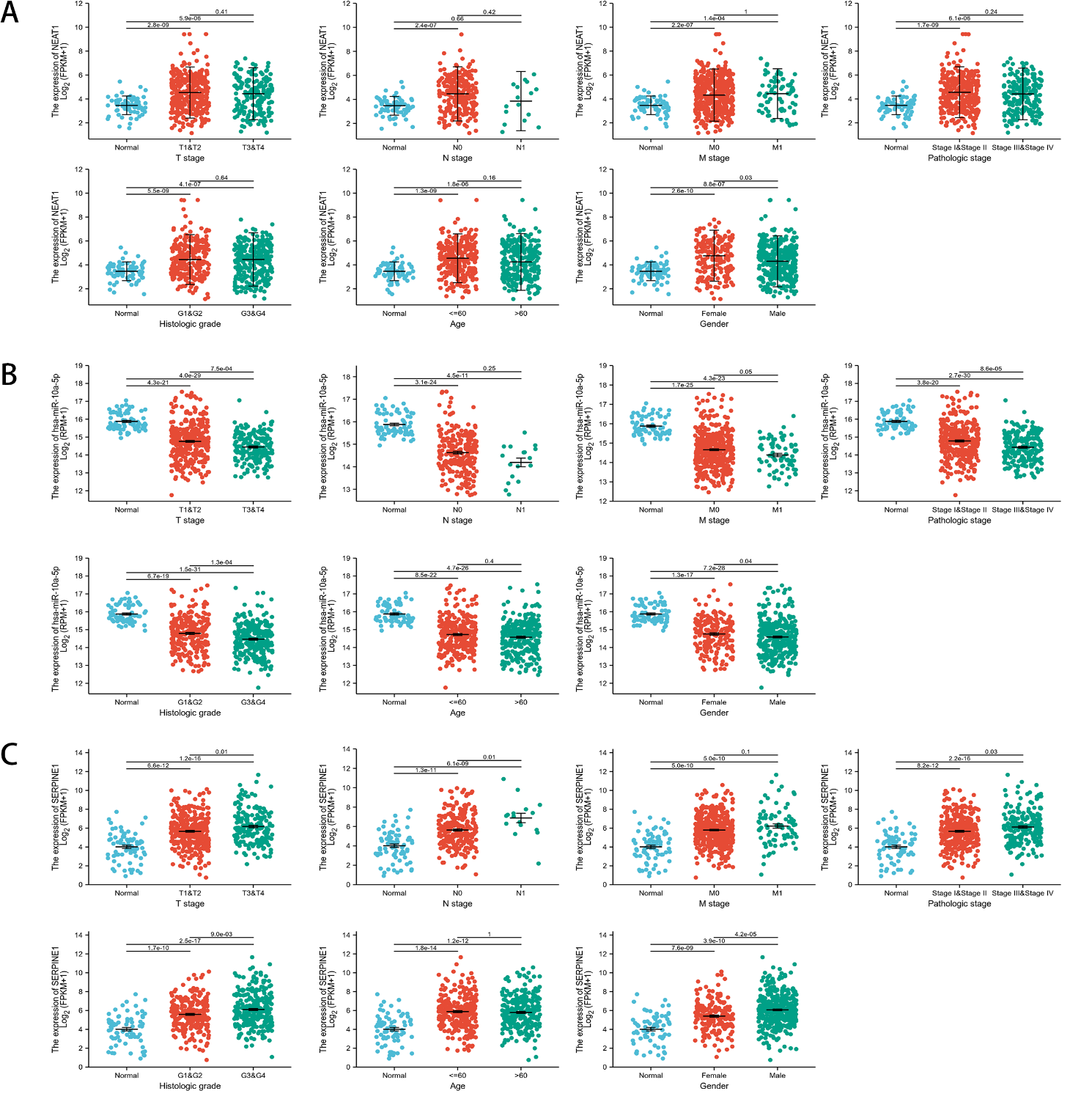
**

**Figure S3. Clinical relevance of NEAT1 (A), miR-10a-5p (B), SERPINE1 (C) in ccRCC patients.**

**
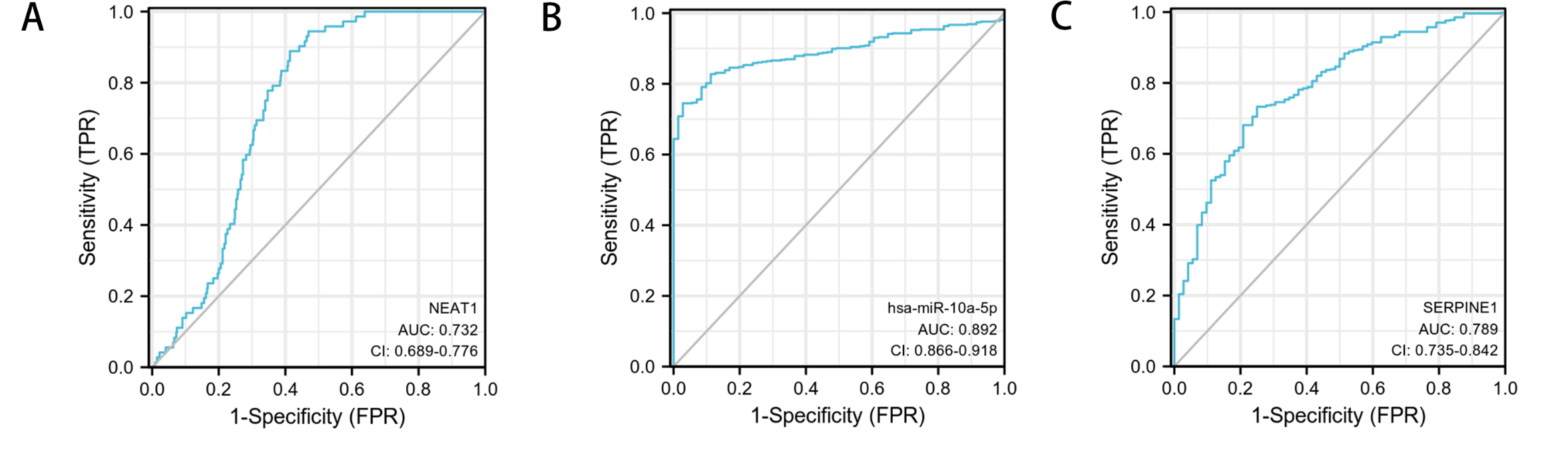
**

**Figure S4. ROC analysis of NEAT1 (A); miR-10a-5p (B); SERPINE1(C).**

**
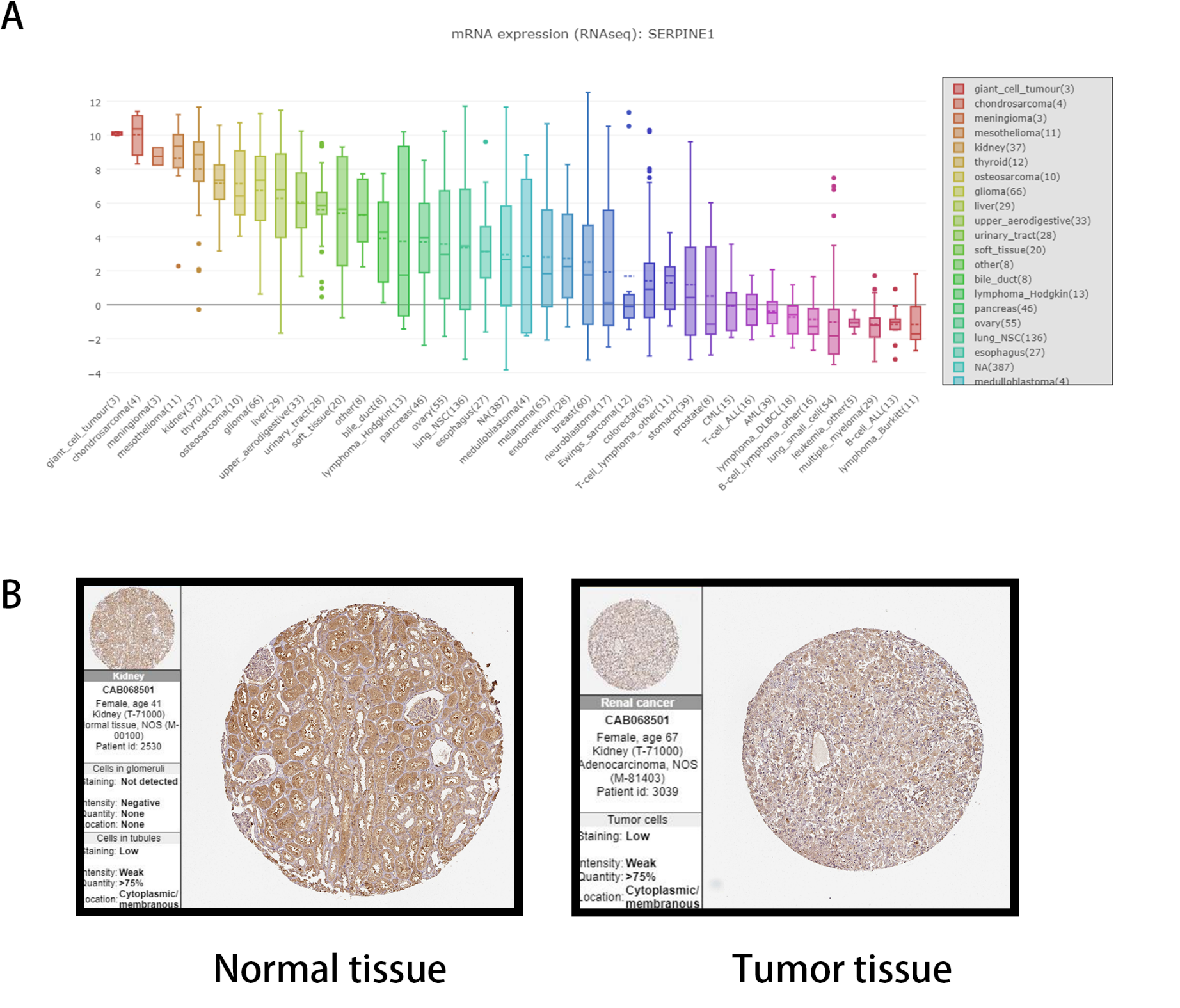
**

**Figure S5. SERPINE1 was aberrantly highly expressed in kidney cancer. (A) mRNA expression in pan-cancer; (B) immunohistochemical analysis of SERPINE1 in normal and tumor tissues.**
