# Supplementary material for: BAP1-Related ceRNA (NEAT1/miR-10a-5p/SERPINE1) Promotes Proliferation and Migration of Kidney Cancer Cells
Source: Front Oncol. 2022 Mar 29;12:852515. doi: 10.3389/fonc.2022.852515 (PMC9004599; doi:10.3389/fonc.2022.852515)
Supplement: Supplementary file 3 [file Table_6.docx]

foldChange=2

padj=0.05

setwd("C:\\Users\\huagb\\Desktop\\TCGA\\01mRNA") #设置工作目录

library("edgeR")

rt=read.table("mRNA_symbol.txt",sep="\t",header=T,check.names=F) #改成自己的文件名

rt=as.matrix(rt)

rownames(rt)=rt[,1]

exp=rt[,2:ncol(rt)]

dimnames=list(rownames(exp),colnames(exp))

data=matrix(as.numeric(as.matrix(exp)),nrow=nrow(exp),dimnames=dimnames)

data=avereps(data)

data=data[rowMeans(data)>1,]

group=c(rep("normal",3),rep("tumor",306)) #按照癌症和正常样品数目修改

design <- model.matrix(~group)

y <- DGEList(counts=data,group=group)

y <- calcNormFactors(y)

y <- estimateCommonDisp(y)

y <- estimateTagwiseDisp(y)

et <- exactTest(y,pair = c("normal","tumor"))

topTags(et)

ordered_tags <- topTags(et, n=100000)

allDiff=ordered_tags$table

allDiff=allDiff[is.na(allDiff$FDR)==FALSE,]

diff=allDiff

newData=y$pseudo.counts

write.table(diff,file="edgerOut.xls",sep="\t",quote=F)

diffSig = diff[(diff$FDR < padj & (diff$logFC>foldChange | diff$logFC<(-foldChange))),]

write.table(diffSig, file="diffSig.xls",sep="\t",quote=F)

diffUp = diff[(diff$FDR < padj & (diff$logFC>foldChange)),]

write.table(diffUp, file="up.xls",sep="\t",quote=F)

diffDown = diff[(diff$FDR < padj & (diff$logFC<(-foldChange))),]

write.table(diffDown, file="down.xls",sep="\t",quote=F)

normalizeExp=rbind(id=colnames(newData),newData)

write.table(normalizeExp,file="normalizeExp.txt",sep="\t",quote=F,col.names=F) #输出所有基因校正后的表达值（normalizeExp.txt）

diffExp=rbind(id=colnames(newData),newData[rownames(diffSig),])

write.table(diffExp,file="diffmRNAExp.txt",sep="\t",quote=F,col.names=F) #输出差异基因校正后的表达值（diffmRNAExp.txt）

heatmapData <- newData[rownames(diffSig),]

#volcano

#pdf(file="vol.pdf")

tiff(file="vol.tiff",width =12,height =12,units ="cm",compression="lzw",bg="white",res=400)

xMax=max(-log10(allDiff$FDR))+1

yMax=12

plot(-log10(allDiff$FDR), allDiff$logFC, xlab="-log10(FDR)",ylab="logFC",

main="Volcano", xlim=c(0,xMax),ylim=c(-yMax,yMax),yaxs="i",pch=20, cex=0.4)

diffSub=allDiff[allDiff$FDR<padj & allDiff$logFC>foldChange,]

points(-log10(diffSub$FDR), diffSub$logFC, pch=20, col="red",cex=0.4)

diffSub=allDiff[allDiff$FDR<padj & allDiff$logFC<(-foldChange),]

points(-log10(diffSub$FDR), diffSub$logFC, pch=20, col="green",cex=0.4)

abline(h=0,lty=2,lwd=3)

dev.off()

#heatmap

hmExp=log10(heatmapData+0.001)

library('gplots')

hmMat=as.matrix(hmExp)

#pdf(file="heatmap.pdf",width=60,height=90)

tiff(file="heatmap.tiff",width =60,height =90,units ="cm",compression="lzw",bg="white",res=400)

par(oma=c(10,3,3,7))

heatmap.2(hmMat,col='greenred',trace="none")

dev.off()

#biocLite("edgeR")

#install.packages("gplots")

foldChange=2

padj=0.05

setwd("C:\\Users\\huagb\\Desktop\\TCGA\\02lncRNA") #设置工作目录

library("edgeR")

rt=read.table("lncRNA_symbol.txt",sep="\t",header=T,check.names=F) #改成自己的文件名

rt=as.matrix(rt)

rownames(rt)=rt[,1]

exp=rt[,2:ncol(rt)]

dimnames=list(rownames(exp),colnames(exp))

data=matrix(as.numeric(as.matrix(exp)),nrow=nrow(exp),dimnames=dimnames)

data=avereps(data)

data=data[rowMeans(data)>1,]

group=c(rep("normal",3),rep("tumor",306)) #按照癌症和正常样品数目修改

design <- model.matrix(~group)

y <- DGEList(counts=data,group=group)

y <- calcNormFactors(y)

y <- estimateCommonDisp(y)

y <- estimateTagwiseDisp(y)

et <- exactTest(y,pair = c("normal","tumor"))

topTags(et)

ordered_tags <- topTags(et, n=100000)

allDiff=ordered_tags$table

allDiff=allDiff[is.na(allDiff$FDR)==FALSE,]

diff=allDiff

newData=y$pseudo.counts

write.table(diff,file="edgerOut.xls",sep="\t",quote=F)

diffSig = diff[(diff$FDR < padj & (diff$logFC>foldChange | diff$logFC<(-foldChange))),]

write.table(diffSig, file="diffSig.xls",sep="\t",quote=F)

diffUp = diff[(diff$FDR < padj & (diff$logFC>foldChange)),]

write.table(diffUp, file="up.xls",sep="\t",quote=F)

diffDown = diff[(diff$FDR < padj & (diff$logFC<(-foldChange))),]

write.table(diffDown, file="down.xls",sep="\t",quote=F)

normalizeExp=rbind(id=colnames(newData),newData)

write.table(normalizeExp,file="normalizeExp.txt",sep="\t",quote=F,col.names=F) #输出所有基因校正后的表达值（normalizeExp.txt）

diffExp=rbind(id=colnames(newData),newData[rownames(diffSig),])

write.table(diffExp,file="diffmRNAExp.txt",sep="\t",quote=F,col.names=F) #输出差异基因校正后的表达值（diffmRNAExp.txt）

heatmapData <- newData[rownames(diffSig),]

#volcano

#pdf(file="vol.pdf")

tiff(file="vol.tiff",width =12,height =12,units ="cm",compression="lzw",bg="white",res=400)

xMax=max(-log10(allDiff$FDR))+1

yMax=12

plot(-log10(allDiff$FDR), allDiff$logFC, xlab="-log10(FDR)",ylab="logFC",

main="Volcano", xlim=c(0,xMax),ylim=c(-yMax,yMax),yaxs="i",pch=20, cex=0.4)

diffSub=allDiff[allDiff$FDR<padj & allDiff$logFC>foldChange,]

points(-log10(diffSub$FDR), diffSub$logFC, pch=20, col="red",cex=0.4)

diffSub=allDiff[allDiff$FDR<padj & allDiff$logFC<(-foldChange),]

points(-log10(diffSub$FDR), diffSub$logFC, pch=20, col="green",cex=0.4)

abline(h=0,lty=2,lwd=3)

dev.off()

#heatmap

hmExp=log10(heatmapData+0.001)

library('gplots')

hmMat=as.matrix(hmExp)

#pdf(file="heatmap.pdf",width=60,height=90)

tiff(file="heatmap.tiff",width =60,height =90,units ="cm",compression="lzw",bg="white",res=400)

par(oma=c(10,3,3,7))

heatmap.2(hmMat,col='greenred',trace="none")

dev.off()

foldChange=2

padj=0.05

setwd("C:\\Users\\huagb\\Desktop\\TCGA\\03miRNA") #设置工作目录

library("edgeR")

rt=read.table("miRNAmatrix.txt",sep="\t",header=T,check.names=F) #改成自己的文件名

rt=as.matrix(rt)

rownames(rt)=rt[,1]

exp=rt[,2:ncol(rt)]

dimnames=list(rownames(exp),colnames(exp))

data=matrix(as.numeric(as.matrix(exp)),nrow=nrow(exp),dimnames=dimnames)

data=avereps(data)

data=data[rowMeans(data)>1,]

group=c(rep("normal",3),rep("tumor",309)) #按照癌症和正常样品数目修改

design <- model.matrix(~group)

y <- DGEList(counts=data,group=group)

y <- calcNormFactors(y)

y <- estimateCommonDisp(y)

y <- estimateTagwiseDisp(y)

et <- exactTest(y,pair = c("normal","tumor"))

topTags(et)

ordered_tags <- topTags(et, n=100000)

allDiff=ordered_tags$table

allDiff=allDiff[is.na(allDiff$FDR)==FALSE,]

diff=allDiff

newData=y$pseudo.counts

write.table(diff,file="edgerOut.xls",sep="\t",quote=F)

diffSig = diff[(diff$FDR < padj & (diff$logFC>foldChange | diff$logFC<(-foldChange))),]

write.table(diffSig, file="diffSig.xls",sep="\t",quote=F)

diffUp = diff[(diff$FDR < padj & (diff$logFC>foldChange)),]

write.table(diffUp, file="up.xls",sep="\t",quote=F)

diffDown = diff[(diff$FDR < padj & (diff$logFC<(-foldChange))),]

write.table(diffDown, file="down.xls",sep="\t",quote=F)

normalizeExp=rbind(id=colnames(newData),newData)

write.table(normalizeExp,file="normalizeExp.txt",sep="\t",quote=F,col.names=F) #输出所有基因校正后的表达值（normalizeExp.txt）

diffExp=rbind(id=colnames(newData),newData[rownames(diffSig),])

write.table(diffExp,file="diffmRNAExp.txt",sep="\t",quote=F,col.names=F) #输出差异基因校正后的表达值（diffmRNAExp.txt）

heatmapData <- newData[rownames(diffSig),]

#volcano

#pdf(file="vol.pdf")

tiff(file="vol.tiff",width =12,height =12,units ="cm",compression="lzw",bg="white",res=400)

xMax=max(-log10(allDiff$FDR))+1

yMax=12

plot(-log10(allDiff$FDR), allDiff$logFC, xlab="-log10(FDR)",ylab="logFC",

main="Volcano", xlim=c(0,xMax),ylim=c(-yMax,yMax),yaxs="i",pch=20, cex=0.4)

diffSub=allDiff[allDiff$FDR<padj & allDiff$logFC>foldChange,]

points(-log10(diffSub$FDR), diffSub$logFC, pch=20, col="red",cex=0.4)

diffSub=allDiff[allDiff$FDR<padj & allDiff$logFC<(-foldChange),]

points(-log10(diffSub$FDR), diffSub$logFC, pch=20, col="green",cex=0.4)

abline(h=0,lty=2,lwd=3)

dev.off()

#heatmap

hmExp=log10(heatmapData+0.001)

library('gplots')

hmMat=as.matrix(hmExp)

#pdf(file="heatmap.pdf",width=60,height=90)

tiff(file="heatmap.tiff",width =60,height =90,units ="cm",compression="lzw",bg="white",res=400)

par(oma=c(10,3,3,7))

heatmap.2(hmMat,col='greenred',trace="none")

dev.off()

use strict;

use warnings;

my %hash=();

open(RF,"diff_lncRNA.txt") or die $!;

while(my $line=<RF>){

chomp($line);

$hash{$line}=1;

}

close(RF);

open(RF,"mircode.txt") or die $!;

open(WF,">lncRNA_mircode.txt") or die $!;

while(my $line=<RF>){

if($.==1){

print WF $line;

next;

}

my @arr=split(/\t/,$line);

my @zeroArr=split(/\|/,$arr[1]);

if(exists $hash{$zeroArr[0]}){

print WF $line;

}

}

close(WF);

close(RF);

use strict;

use warnings;

my %hash=();

open(RF,"diff_miRNA.txt") or die $!;

while(my $line=<RF>){

chomp($line);

$line=~s/^\s+|\s+$//g;

$hash{$line}=1;

}

close(RF);

my %repHash=();

open(RF,"lncRNA_mircode.txt") or die $!;

open(WF,">lncRNA_miRNA.txt") or die $!;

print WF "lncRNA\tmiRNA\n";

while(my $line=<RF>){

my @arr=split(/\t/,$line);

my @threeArr=split(/\//,$arr[3]);

$threeArr[0]=~s/miR\-//g;

foreach my $mirna(@threeArr){

if(exists $hash{"hsa-mir-$mirna"}){

unless(exists $repHash{"$arr[1]\thsa-mir-$mirna"}){

print WF "$arr[1]\thsa-mir-$mirna\n";

$repHash{"$arr[1]\thsa-mir-$mirna"}=1;

}

}

}

}

close(WF);

close(RF);

use strict;

use warnings;

my %hash=();

open(RF,"DEmiRNA.txt") or die $!;

while(my $line=<RF>){

chomp($line);

$hash{$line}=1;

}

close(RF);

open(RF,"starBase_miRNA.txt") or die $!;

open(WF,">miRNA.txt") or die $!;

while(my $line=<RF>){

if($.==1){

print WF $line;

next;

}

my @arr=split(/\t/,$line);

my @zeroArr=split(/\|/,$arr[0]);

if(exists $hash{$zeroArr[0]}){

print WF $line;

}

}

close(WF);

close(RF);

#split函数讲字符串转换为数组

#$. 前一次读的文件句柄的当前行号

#exists一般是用来测试hash表中是否存在一个变量

#chomp()函数去掉字符串结尾的换行符

use strict;

use warnings;

my %miHash=();

open(RF,"miRNA.txt") or die $!;

while(my $line=<RF>){

chomp($line);

$line=~s/^\s+|\s+$//g;

$miHash{$line}=1;

}

close(RF);

my %hash=();

my @files=glob("*.tsv");

my @dbs=();

foreach my $file(@files){

my $db=$file;

$db=~s/\.tsv//g;

push(@dbs,$db);

open(RF,"$file") or die $!;

while(my $line=<RF>){

chomp($line);

my @arr=split(/\t/,$line);

if(exists $miHash{$arr[0]}){

my $mirnaGene="$arr[0]\t$arr[1]";

${$hash{$mirnaGene}}{$db}=1;

}

}

close(RF);

}

open(WF,">result.xls") or die $!;

print WF "miRNA\tGene\t" . join("\t",@dbs) . "\tSum\n";

foreach my $key(keys %hash){

my $outLine=$key;

my $sum=0;

foreach my $db(@dbs){

if(exists ${$hash{$key}}{$db}){

$sum++;

$outLine=$outLine . "\t1";

}

else{

$outLine=$outLine . "\t0";

}

}

if($sum>=3){

print WF $outLine . "\t$sum\n";

}

}

close(WF);

#install.packages("VennDiagram")

setwd("C:\\Users\\huagb\\Desktop\\TCGA\\05miRNA_Target\\03mRNA_Venny")

files=dir()

files=grep("txt",files,value=T)

targetList=list()

for(i in 1:length(files)){

inputFile=files[i]

rt=read.table(inputFile,header=F)

header=unlist(strsplit(inputFile,"\\.|\\-"))

targetList[[header[1]]]=as.vector(rt[,1])

uniqLength=length(unique(as.vector(rt[,1])))

print(paste(header[1],uniqLength,sep=" "))

}

library(VennDiagram)

venn.diagram(targetList,filename="venny.tiff",imagetype = "tiff",

fill=rainbow(length(targetList)),cat.cex=0.6)

intersectGenes=Reduce(intersect,targetList)

write.table(file="target.xls",intersectGenes,sep="\t",quote=F,col.names=F,row.names=F)

use strict;

use warnings;

my %hash=();

open(RF,"DEmRNA.txt") or die $!;

while(my $line=<RF>){

chomp($line);

$hash{$line}=1;

}

close(RF);

open(RF,"miRNA_Target.txt") or die $!;

open(WF,">miRNA_mRNA.txt") or die $!;

while(my $line=<RF>){

if($.==1){

print WF $line;

next;

}

my @arr=split(/\t/,$line);

my @zeroArr=split(/\|/,$arr[1]);

if(exists $hash{$zeroArr[0]}){

print WF $line;

}

}

close(WF);

close(RF);

#install.packages('survival')

#source("https://bioconductor.org/biocLite.R")

#biocLite("qvalue")

setwd("C:\\Users\\huagb\\Desktop\\TCGA\\07Survival\\02lncRNA_survival\\survival")

outTab=data.frame()

picDir="picture"

dir.create(picDir)

library(survival)

library(qvalue)

rt=read.table("tumor.time.txt",header=T,sep="\t",row.names=1,check.names=F)

rt[,"futime"]=rt[,"futime"]/365

for(i in colnames(rt[,3:ncol(rt)])){

cox <- coxph(Surv(futime, fustat) ~ rt[,i], data = rt)

coxSummary = summary(cox)

coxP=coxSummary$coefficients[,"Pr(>|z|)"]

med=median(rt[,i])

if(med!=0){

a=rt[,i]>med

rt1=rt[a,]

b=setdiff(rownames(rt),rownames(rt1))

rt2=rt[b,]

n1=nrow(rt1)

n2=nrow(rt2)

surTab1=summary(survfit(Surv(futime, fustat) ~ 1, data = rt1))

surTab2=summary(survfit(Surv(futime, fustat) ~ 1, data = rt2))

medianTab1=surTab1$table

medianTab2=surTab2$table

diff=survdiff(Surv(futime, fustat) ~a,data = rt)

fit <- survfit(Surv(futime, fustat) ~ a, data = rt)

pValue=1-pchisq(diff$chisq,df=1)

outTab=rbind(outTab,cbind(gene=i,coxSummary$coefficients,coxSummary$conf.int,KM=pValue,

H_med=medianTab1["median"],H_0.95LCL=medianTab1["0.95LCL"],H_0.95UCL=medianTab1["0.95UCL"],

L_med=medianTab2["median"],L_0.95LCL=medianTab2["0.95LCL"],L_0.95UCL=medianTab2["0.95UCL"]))

pval=0

if(pValue<0.05){

pval=signif(pValue,4)

pval=format(pval, scientific = TRUE)

}else{

pval=round(pValue,3)

}

if(pValue<0.05){

geneName=unlist(strsplit(i,"\\|",))[1]

tiffFile=paste(geneName,".survival.tiff",sep="")

outTiff=paste(picDir,tiffFile,sep="\\")

tiff(file=outTiff,width = 15,height = 15,units ="cm",compression="lzw",bg="white",res=600)

plot(fit, col=c("blue","red"), xlab="Time (years)", ylab="Overall survival",

main=paste(geneName,"(p=",pval, ")", sep=""),mark.time=T,ylim=c(0,1.1),

lwd = 2, cex.main=1.3, cex.lab=1.2, cex.axis=1.2, font=1.2)

legend("topright", c(paste("Low expression"),

paste("High expression")),

col=c("blue","red"), bty="n", lwd = 2, cex=0.8)

dev.off()

}

}

}
